# Openness to “Sugar Relationships” Reflects Personality and Emotional Vulnerabilities in a Representative Sample of Hungarian Women

**DOI:** 10.1007/s10508-025-03389-8

**Published:** 2026-03-10

**Authors:** Norbert Meskó, Béla Birkás, András N. Zsidó

**Affiliations:** 1 Department of Cognitive and Evolutionary Psychology, Faculty of Humanities and Social Sciences, Institute of Psychology Pécs, University of Pécs, Ifjúság Utca 6, Pécs, 7624 Hungary; 2 Department of Behavioral Sciences, Medical School, University of Pécs, Pécs, Hungary

**Keywords:** Sugar relationships, Personality functioning, Emotion regulation, Early maladaptive schemas, Female sexuality

## Abstract

Openness to sugar relationships—arrangements in which companionship or sexual intimacy is exchanged for material benefits within loosely structured, mutually negotiated agreements—has garnered growing attention in psychological research. However, little is known about the underlying personality and emotion regulation patterns associated with such attitudes. In this preregistered study, we examined whether early maladaptive schemas (EMS), personality functioning (LPFS-BF 2.0), and cognitive emotion regulation strategies (CERQ) predicted openness to sugar relationships (ASR) in a large, demographically stratified sample of young Hungarian women (*N* = 500; ages 18–35). Participants completed validated self-report questionnaires. General linear models revealed that ASR scores were significantly predicted by impaired personality functioning and maladaptive emotion regulation strategies. Furthermore, these vulnerabilities were strongly associated with elevated EMS scores. Structural equation modeling supported a mediation model in which EMS indirectly predicted ASR via emotion regulation and personality functioning. These findings suggest that openness to sugar relationships may reflect not only strategic or permissive attitudes but also deeper vulnerabilities in self and relational functioning. By integrating dimensional models of personality pathology and emotion regulation with emerging research on transactional sexual attitudes, this study highlights the psychological underpinnings of receptivity to sugar relationships. The results underscore the need to view such relational preferences within a broader developmental and psychological framework rather than moral or economic lenses. This is the first study to establish these associations using a representative sample of young women, contributing to a more nuanced understanding of individual differences in sexual attitudes and behaviors.

**Supplementary Information:**

The online version contains supplementary material available at 10.1007/s10508-025-03389-8.

## Introduction

### Sugar Relationships

Sugar relationships represent a contemporary form of transactional exchange in which companionship or sexual intimacy is traded for material resources such as money, gifts, or social capital (Nayar, 2017; Nelson, 1993). These arrangements are most commonly formed between older men (“sugar daddies”) and younger women (“sugar babies”), although male–male and female–younger male configurations also occur. In all cases, the dynamic involves an older partner with economic or professional resources and a younger partner offering companionship and/or sexual intimacy (Meskó et al., 2024). Although often facilitated by specialized online platforms, these relationships vary widely in structure, intimacy, and duration, ranging from explicitly sexual arrangements to more ambiguous forms of companionship (Meskó et al., 2025; Ojebode et al., 2010). Similar patterns have been documented in qualitative studies from diverse cultural contexts, demonstrating that transactional and sugar-like relationships can also involve emotional intimacy, curiosity-driven engagement, or companionship-oriented exchanges (Scull, 2020; Scull, 2022; Shefer et al., 2011; Strebel et al., 2013; Selikow & Mbulaheni, 2013).

Despite ongoing debates regarding whether sugar relationships constitute a form of sex work, prior research has shown that these arrangements reflect a complex interplay of economic motives, sexual strategies, and relational expectations (Láng et al., 2021; Nelson, 1993). This complexity is echoed in broader qualitative and cross-cultural work on transactional intimacy, which shows that economic exchange, strategic partner selection, and emotional negotiation often coexist in diverse forms of compensated relationships (e.g., Scull, 2020; Shefer et al., 2011). Consequently, sugar relationships are increasingly conceptualized as psychologically meaningful mating phenomena situated along a continuum of sex-for-resources exchanges in human sexual behavior (Birkás et al., 2020; Meskó et al., 2025).

While sugar relationships are frequently examined in respect to moral (Palomeque Recio, 2022), economic (Mixon, 2019), or legal aspects (Motyl, 2012), their psychological underpinnings have only recently become the focus of empirical research (Birkás et al., 2020; Ipolyi et al., 2021; Láng et al., 2021). Attitudes toward such arrangements are shaped by enduring individual differences in mating strategies, relational expectations, and value systems regarding intimacy and exchange (Nayar, 2017; Otto et al., 2021). Understanding these attitudes is crucial, not only because they influence behavioral intentions in romantic contexts, but also because they are grounded in stable cognitive-affective dispositions. Within this framework, the construct of openness to sugar relationships has emerged as a theoretically relevant and psychometrically valid attitudinal domain (Birkás et al., 2020; Láng et al., 2021). This construct measures the extent to which individuals endorse or accept such arrangements as legitimate forms of romantic or sexual involvement. As such, it offers a psychologically relevant approach for investigating modern relationship norms, especially among younger individuals navigating a post-conventional sexual marketplace (Meskó et al., 2024). Importantly, this openness may also reflect evolutionarily shaped strategies for resource exchange in mating—an embedded logic of sexual-economic reciprocity, persistent across diverse cultural contexts (Conroy-Beam & Buss, 2019; Meskó et al., 2024, 2025).

Emerging empirical evidence regarding the psychological correlates of openness to sugar relationships indicates that the construct is not merely a sociocultural artifact but is systematically associated with personality traits and relational dispositions. For instance, individuals scoring higher on this dimension have been shown to exhibit elevated levels of borderline personality features (Láng et al., 2021) as well as traits from the Dark Triad, particularly narcissism and Machiavellianism (Birkás et al., 2020). These findings suggest a relational orientation marked by reduced affective commitment, strategic interpersonal control, and increased sensitivity to resource-based exchanges in romantic contexts.

Furthermore, openness to sugar relationships has been linked to distinct motivational profiles, including short-term mating orientation (Meskó et al., 2025) and extrinsic motivation, especially among younger individuals (Ipolyi et al., 2021). Notably, attachment avoidance has been found to moderate the relationship between extrinsic motivation and acceptance of sugar relationships, implying that the regulation of interpersonal closeness may influence attitudes toward transactional sexual exchanges (Ipolyi et al., 2021). Collectively, prior findings support the notion that openness to sugar relationships constitutes a psychologically meaningful disposition embedded within broader personality systems and motivational hierarchies. However, its associations with deeper intrapsychic structures—such as early maladaptive schemas and habitual emotion regulation strategies—remain underexplored. The present study aims to address this gap.

### Early Maladaptive Schemas and Their Relevance to Sugar Relationship Attitudes

The concept of EMS, introduced by Young et al. (2003), refers to pervasive and self-defeating cognitive-emotional patterns that originate in childhood and persist throughout life. EMS develop in response to unmet core emotional needs, typically arising from adverse childhood experiences such as emotional neglect, abuse, or abandonment. Schemas represent enduring frameworks encompassing memories, emotions, bodily sensations, and cognitions and constitute deeply embedded cognitive-affective structures that shape self-perception, interpersonal expectations, and relational functioning. Among the 18 EMS identified in schema theory, those within the disconnection and rejection domain (e.g., abandonment, mistrust/abuse, emotional deprivation, and defectiveness/shame) are particularly relevant to relational patterns, as they involve persistent expectations that one’s needs for security, stability, empathy, and respect will not be met in relationships (Young et al., 2003).

Large body of empirical research has linked EMS to various maladaptive outcomes, including depression (Harris & Curtin, 2002), anxiety (Wright et al., 2009), personality disorders (Jovev & Jackson, 2004), and risky interpersonal behaviors such as self-harm and revictimization (Crawford & Wright, 2007; Messman-Moore & Coates, 2007). More specifically, recent evidence suggests that EMS, especially within the disconnection and rejection domain, may mediate the relationship between childhood abuse and risky sexual behavior. Roemmele and Messman-Moore (2011) found that schemas such as abandonment, defectiveness/shame, and mistrust/abuse significantly predicted both the number of sexual partners and the likelihood of engaging in unprotected or non-intimate sexual encounters. These findings support the idea that EMS may function as cognitive-affective vulnerabilities, motivating individuals to seek relational validation or exert control through sex, even in ways that compromise emotional or physical safety.

Sugar relationships often circumvent the expectations of emotional intimacy and mutual vulnerability characteristic of committed partnerships, emphasizing instead instrumental motives and resource-based exchange. Individuals with heightened EMS in the disconnection domain may be drawn to such relational formats as a means of managing fears of abandonment or unworthiness while simultaneously preserving interpersonal distance and control. Accordingly, sugar relationships may reflect a compensatory, but maladaptive relational strategy for individuals whose early experiences have undermined trust and attachment security. Considering these conceptual connections between EMS content and the dynamics of sugar relationships, the present study investigates whether these schemas are associated with increased openness to such arrangements among young women.

### Personality Functioning and Vulnerabilities in Intimate Relationships

The introduced in Section III of the DSM-5 (American Psychiatric Association, 2013), reconceptualizes personality pathology as impairments in self and interpersonal functioning. According to this model, the core of all personality disorders lies not only in specific maladaptive traits but also in disruptions of fundamental personality functions—namely identity, self-direction, empathy, and intimacy. Impaired self-functioning commonly manifests as identity diffusion, affective dysregulation, and incoherent goal pursuit, while interpersonal dysfunction is marked by difficulties with empathy, mutuality, and sustaining intimate relationships (Major et al., 2024). These impairments are assessed along a severity continuum using the Level of Personality Functioning Scale (LPFS), which has been operationalized through both clinical interviews and self-report instruments (Morey et al., 2011; Weekers et al., 2019). The LPFS-Brief Form 2.0 (LPFS-BF 2.0) provides a concise, psychometrically validated tool for assessing dysfunctions in self- and interpersonal domains and has demonstrated strong associations with a range of personality problems and clinical syndromes (Bach & Anderson, 2018; Weekers et al., 2019).

Nevertheless, LPFS-based assessments also offer valuable insights into subclinical relational tendencies and vulnerabilities in the general population. Individuals with lower levels of personality functioning frequently exhibit chaotic interpersonal styles, emotional lability, and difficulties regulating closeness and autonomy—features that may predispose them to relational formats characterized by limited emotional involvement or opportunities for strategic interpersonal control. Research on sex workers and women involved in transactional sex highlights the role of impaired identity integration, compromised relational capacity, and difficulties in emotion regulation as underlying mechanisms contributing to engagement in such exchanges (e.g., Tyler, 2009; Westen et al., 2011). These findings provide a theoretical rationale for examining whether impairments in personality functioning are predictive of receptivity to resource-based sexual or romantic arrangements, such as sugar relationships.

Individuals with diminished capacities for affect regulation, coherent self-experience, and secure attachment may perceive sugar relationships as safer and more predictable alternatives to emotionally demanding conventional partnerships. Moreover, transactional relationships may serve as compensatory strategies that allow for controlled proximity and predefined expectations, thereby reducing the risk of rejection or emotional enmeshment. Supporting this interpretation, recent evidence from studies comparing sex workers to matched control groups indicates that these individuals tend to show higher levels of Machiavellianism, earlier substance use, faster life history strategies, and greater endorsement of short-term mating attitudes (Cabrera, 2023; Edlund et al., 2023). These findings reinforce the view that non-normative relational choices—whether sex work or openness to transactional intimacy—are not merely pragmatic but also reflect underlying psychological configurations rooted in personality functioning.

### Emotion Regulation and Sexual Decision-Making

Emotion regulation (ER) refers to the set of psychological processes through which individuals influence the experience, expression, and modulation of their emotional states (Gross, 1998). Effective emotion regulation is essential for psychological well-being and interpersonal functioning, and it plays a particularly important role in shaping women’s sexual behavior, satisfaction, and intimacy (Dubé et al., 2020). ER strategies are typically categorized as adaptive (e.g., reappraisal, acceptance) or maladaptive (e.g., rumination, suppression, avoidance), with the latter consistently associated with negative sexual outcomes. Studies indicate that women who rely on disengagement or perseverative strategies are more likely to report sexual dissatisfaction, reduced desire, and heightened relational conflict (Dubé et al., 2020; Velten & Margraf, 2017). In contrast, greater emotional clarity, mindfulness, and cognitive flexibility have been linked to increased sexual satisfaction and better relational adjustment.

Beyond its role in sexual well-being, emotion regulation also influences risky sexual behavior, including casual and transactional sex. Women with poor ER skills may engage in sexual activity to manage negative affect, enhance self-worth, or regain a sense of control (Thompson & Kingree, 2010; Weiss et al., 2011). Emotionally avoidant or dysregulated individuals often prioritize external validation or instrumental rewards in sexual decision-making, especially in contexts marked by power imbalances or attachment insecurity. In such context, transactional sexual encounters may serve as both a coping mechanism and a form of agency within emotionally constrained relational environments (Dubé et al., 2020). Research on sex workers and trauma-exposed women further demonstrates that sexual behavior is often part of emotional regulation strategies in stressful situations (van Berlo & Broaddus, 2005; Zahm et al., 2021).

### Research Objectives and Hypotheses

Building on this literature, the present study examines whether specific emotion regulation styles—as measured by the Cognitive Emotion Regulation Questionnaire—predict openness to sugar relationships among young adult women. We hypothesize that young women who habitually employ maladaptive ER strategies may be more receptive to sugar relationships, as the negotiated and exchange-based structure of such arrangements can be perceived—at least initially—as offering clearer expectations and more controllable relational boundaries, even if empirical evidence shows that these relationships can also involve unpredictability and emotional complexity (e.g., Palomeque Recio, 2022; Scull, 2020). From this perspective, the idea of sugar relationships may appeal to individuals who perceive conventional romantic involvement as emotionally ambiguous, vulnerable, or demanding in terms of regulatory effort. Conversely, individuals with more adaptive ER capacities may be less inclined to endorse relational formats that they perceive as offering fewer opportunities for mutuality or emotional reciprocity, while recognizing that such qualities vary substantially across both commodified and non-commodified relationships.

**Fig. 1:**
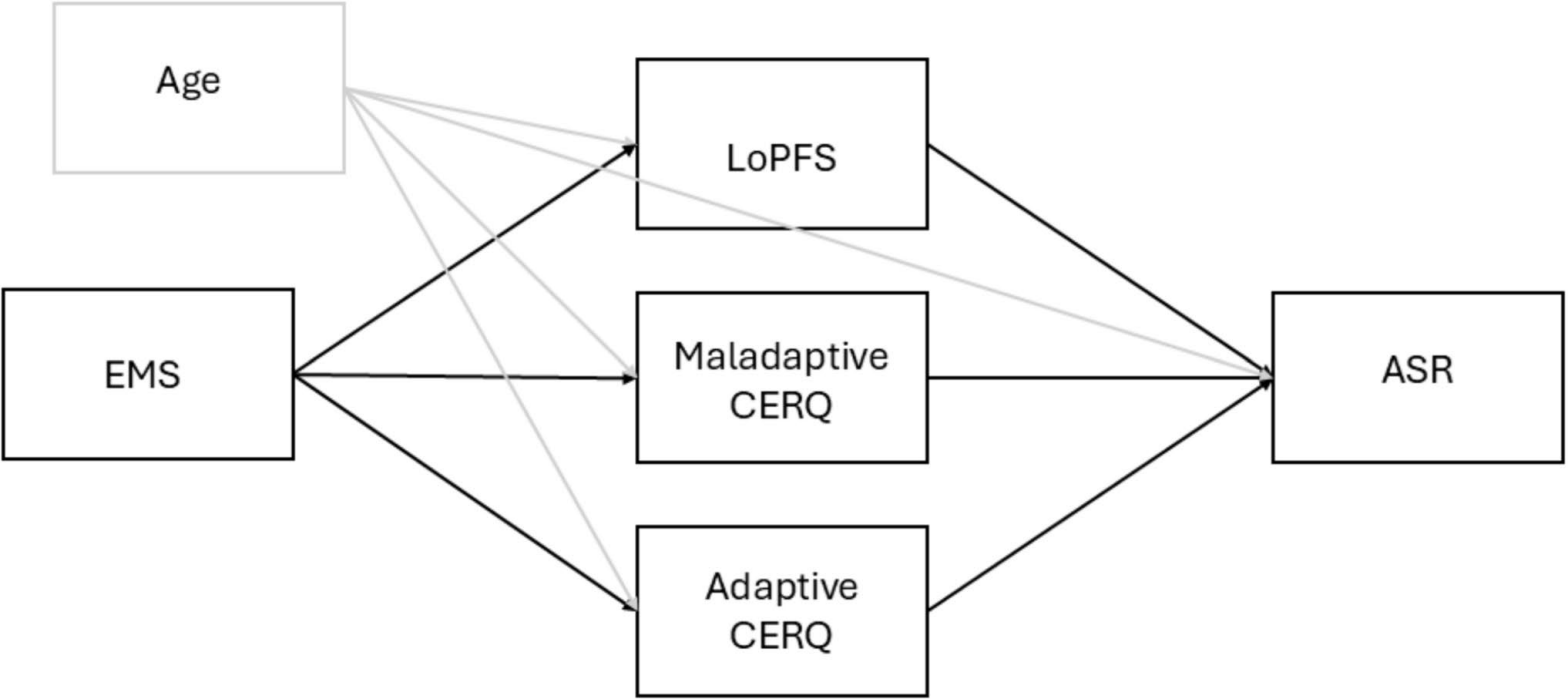
Hypothetical model of psychological factors associated with openness to sugar relationships (ASR). Based on theoretical frameworks and preregistered hypotheses, the model proposes that early maladaptive schemas (EMS) influence ASR indirectly via two mediators: impairments in personality functioning (LoPFS) and the use of maladaptive cognitive emotion regulation strategies (CERQ). Age is included as a covariate. Solid lines indicate hypothesized direct paths; the dashed line represents a covariate effect not derived from theory

Building on these interrelated domains, we propose a structural model in which early maladaptive schemas (EMS) serve as distal cognitive-emotional vulnerabilities that shape relational attitudes indirectly through two key psychological mechanisms: impairments in personality functioning (LPFS) and maladaptive cognitive emotion regulation strategies (CERQ). In this model, EMSs are expected to predict both LPFS and maladaptive CERQ, which in turn predict openness to sugar relationships (ASR). This mediational framework integrates dimensional models of personality pathology and emotion regulation into a broader explanatory system, linking early psychological structures to contemporary relational attitudes (see Fig. 1 for a schematic representation of the model).

The present study aims to examine the psychological correlates of openness to sugar relationships among young adult women. While previous research has investigated how this construct relates to personality traits such as narcissism, Machiavellianism, and borderline tendencies (Birkás et al., 2020; Ipolyi et al., 2021; Láng et al., 2021), relatively little is known about its associations with deeper cognitive and emotional structures. Drawing on schema theory, contemporary models of personality functioning, and affect regulation frameworks, the current study focuses on three psychological domains that may be particularly relevant for understanding individual differences in openness to sugar relationships: early maladaptive schemas, impairments in personality functioning, and cognitive emotion regulation strategies.

These hypotheses are embedded within a broader structural framework in which early maladaptive schemas exert indirect effects on openness to sugar relationships via two mediating mechanisms: impaired personality functioning and maladaptive emotion regulation. This model integrates prior theoretical insights and informs both the analytic strategy and interpretation of findings.

We propose that higher openness to sugar relationships reflects not only attitudinal permissiveness but also underlying personality vulnerabilities and emotion regulation tendencies. Specifically, we hypothesize the following:

- H1: Openness to sugar relationships is positively associated with the presence of early maladaptive schemas.
- H2: Openness to sugar relationships is positively associated with impairments in personality functioning.
- H3: Openness to sugar relationships is positively associated with the use of maladaptive cognitive emotion regulation strategies.
- H4: The associations between early maladaptive schemas and openness to sugar relationships are mediated by impairments in personality functioning and the use of maladaptive cognitive emotion regulation strategies.

By systematically testing these hypotheses in a demographically stratified sample of Hungarian women aged 18 to 35, this study seeks to contribute to a more nuanced understanding of the psychological foundations of transactional relationship acceptance in contemporary mating contexts.

**Fig. 2:**
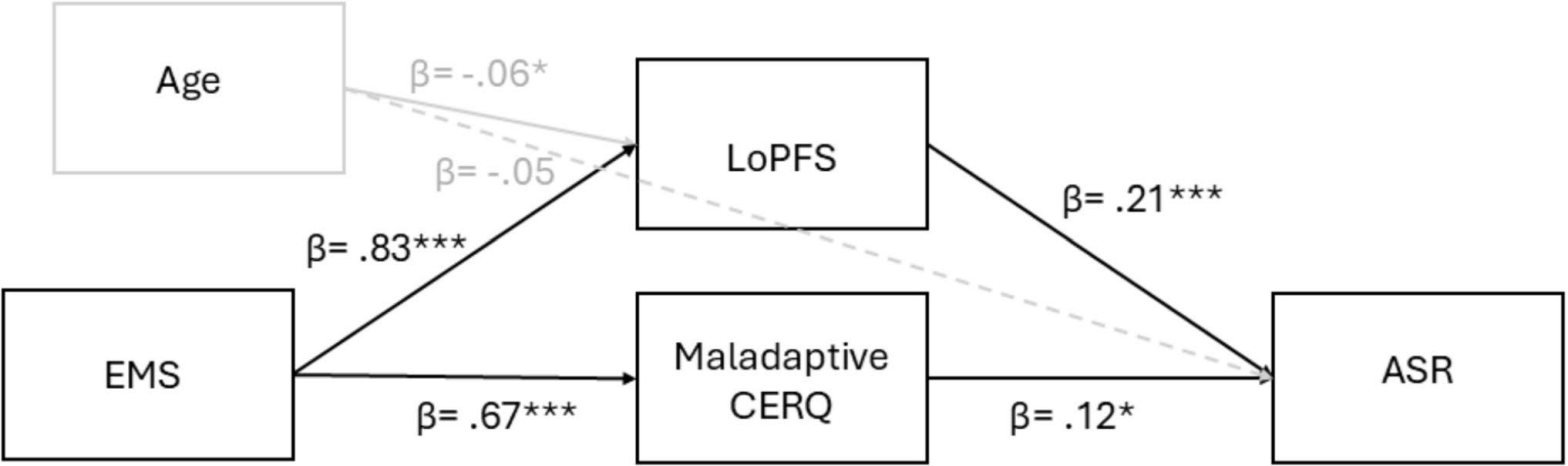
Structural equation model tested in the current study. This empirically trimmed model includes only the significant predictors identified through preliminary GLMs. Statistically significant pathways are shown in black (*p* < .05, *p* < .01, *p* < .001); nonsignificant paths are shown in gray. All estimates are standardized. CERQ = cognitive emotion regulation strategies; LoPFS = level of personality functioning; EMS = early maladaptive schemas

Although the present study was preregistered with a theoretically grounded model (see Fig. 1), the exact structural relationships among variables were not fully specified in advance. Given the exploratory nature of this modeling approach, preliminary general linear models (GLMs) were conducted to identify significant predictors before specifying the final structural equation model (see Fig. 2). This two-step analytic strategy allowed us to first confirm associations among conceptual domains (schemas, personality functioning, and emotion regulation) and then estimate a trimmed model containing only statistically supported pathways. The hypothesized direction of effects—from early maladaptive schemas to openness to sugar relationships, via intrapsychic and regulatory mechanisms—was theoretically justified, but specific path strengths and mediational patterns were determined empirically.

## Method

### Participants and Procedure

The minimum sample size required for the general linear model (GLM) analysis was estimated using a conservative approach (*f* = 0.10, *power* = .95, *α* = .05, *df* = 11) with the pwr package for R (Champely, 2020). This analysis indicated a required sample size of *N* = 200. For the structural equation modeling (SEM) analysis, sample size was estimated based on *RMSEA* = .06, desired power > .80, and *α* = .05, using the web-based interface of the semPower package for R (Moshagen & Bader, 2023), which yielded a recommended minimum of *N* = 350. To enhance statistical precision and enable stratified sampling with population-level representativeness, a larger final sample of 500 participants was collected. As specified in our preregistration, we aimed to collect data from at least 500 participants to ensure sufficient statistical power and allow for stratified sampling; this also explains why the final sample size exceeded the minimum thresholds indicated by the power analyses.

Data collection was conducted in collaboration with a professional public opinion research company based in Hungary. Participants were recruited through the agency’s national online research panel and completed the survey in December 2024. They responded to an anonymous, self-administered questionnaire package via a secure online platform. All participants were young adult Hungarian women between the ages of 18 and 35 years (*M* = 27.56, SD = 4.80).

Although the present research did not assess whether participants had ever engaged in sugar or other transactional relationships, this reflects the intentional conceptual focus of the study rather than a limitation. Our aim was to examine openness to sexual-economic exchange (Meskó, 2025) as a distinct psychological construct—an attitudinal disposition that exists independently of actual behavioral involvement in sugar arrangements. Recent work using the Acceptance of Sugar Relationships Scale (ASR; Birkás et al., 2020) suggests that this openness forms a coherent and meaningful cognitive-affective domain, which shows systematic associations with personality functioning, emotion regulation, and relational motivations. Investigating openness, rather than actual participation, allows us to identify the psychological mechanisms that shape receptivity to transactional intimacy at a broader population level, without restricting analyses to the relatively small subset of individuals who enter sugar relationships. This conceptual distinction represents a central contribution of the present study.

The final sample was stratified to reflect the national distribution of Hungarian women in this age group across key demographic dimensions: educational attainment, geographic region, and settlement type. Regarding education, 7.7% (*n* = 38) of participants had completed primary education or less, 11.4% (*n* = 57) had vocational training, 51.4% (*n* = 257) had completed secondary education (e.g., high school, technical college), and 29.5% (*n* = 148) held a college or university degree. Regarding settlement type, 19.4% (*n* = 97) resided in the capital, 54.0% (*n* = 270) in towns or cities, and 26.6% (*n* = 133) in villages or rural areas. Regional distribution was as follows: 30.6% (*n* = 153) from Central Hungary (including Budapest), 27.4% (*n* = 137) from the Transdanubian regions, and 42.0% (*n* = 210) from Northern Hungary and the Great Plain regions.

Although all participants were assigned female at birth, gender identity was also assessed: 98.6% (*n* = 493) identified as women, 0.3% (*n* = 2) as men, 0.3% (*n* = 2) as non-binary, and 0.8% (*n* = 4) selected “other.” In terms of relationship status, 98.6% (*n* = 493) reported being in a committed romantic relationship, while 1.4% (*n* = 7) were single, separated, or divorced.

Participants were members of a national online research panel and received non‑monetary compensation, such as redeemable points, vouchers, or charitable donation opportunities, in accordance with standard panel practices. All participants provided written informed consent prior to data collection and were informed that they could decline to answer any question or withdraw from the study at any time without consequence.

### Measures

#### Openness to Sugar Relationships

Openness to sugar relationships was assessed with the Acceptance of Sugar Relationships in Young Women and Men Scale (ASR-YWMS; Birkás et al., 2020). The scale defines a sugar relationship as a transactional sexual arrangement in which an older and wealthier partner (sugar daddy/mommy) provides material resources to a younger partner (sugar baby/boy) in exchange for companionship. Partners usually meet to spend leisure time together, with sexual activity occurring only if both parties provide consent. The scale consists of eight items rated on a 7-point Likert scale ranging from 1 (absolutely disagree) to 7 (absolutely agree), with higher scores indicating greater openness to sugar relationships. Example items include: “If I knew I would not incur negative judgment or consequences, I would like to try a sugar relationship,” and “If it would benefit my career, I would think about engaging in a sugar relationship.” In the current study, the internal consistency of the scale was excellent (McDonald’s *ω* = .93). The Hungarian version was identical to the validated version developed by Birkás et al. (2020); no modifications were made to the content or response format.

#### Early Maladaptive Schemas

EMS were assessed using the Young Schema Questionnaire—Short Form, version 3 (YSQ-S3; Young, 2005; Hungarian version: Unoka et al., 2004). This instrument includes 90 self-report items measuring 18 distinct schemas, theorized to reflect pervasive, dysfunctional cognitive-emotional patterns formed in early life. Respondents rated each item on a 6-point Likert scale ranging from 1 (completely untrue of me) to 6 (describes me perfectly). Although the YSQ-S3 is often analyzed at the level of individual schemas, the present study used the total EMS score—summing all item responses—to capture general cognitive-emotional vulnerability, in line with previous research (e.g., Kriston et al., 2013; Shorey et al., 2011). This approach enabled a global assessment of maladaptive schema endorsement. The internal consistency of the total score was excellent in our sample (McDonald’s *ω* = .98).

#### Personality Functioning

Personality functioning was measured using the Level of Personality Functioning Scale – Brief Form 2.0 (LPFS-BF 2.0; Weekers et al., 2019; Hungarian version: Láng & Birkás, 2023). This 12-item self-report measure assesses impairments in self- and interpersonal functioning, consistent with Criterion A of the DSM-5 Alternative Model for Personality Disorders. Items are rated on a 4-point Likert scale ranging from 1 (completely untrue) to 4 (completely true), with higher scores reflecting greater dysfunction. Although the LPFS-BF 2.0 can be interpreted along two dimensions (self and interpersonal functioning), a unidimensional total score was used in this study by summing all items. This approach aligns with prior research aiming to capture global personality dysfunction in a concise format (e.g., Bach & Anderson, 2018; Zimmermann et al., 2020). Internal consistency of the total score was excellent (McDonald’s *ω* = .89).

#### Cognitive Emotion Regulation Strategies

Cognitive emotion regulation was assessed using the short version of the Cognitive Emotion Regulation Questionnaire (CERQ-short; Garnefski & Kraaij, 2006). The 18-item version includes nine subscales from the original CERQ (Garnefski et al., 2001), each represented by two items. These subscales reflect distinct cognitive coping strategies in response to negative or stressful events and are grouped into adaptive (e.g., acceptance, positive reappraisal, planning) and maladaptive (e.g., rumination, catastrophizing, self-blame) strategies. Items are rated on a 5-point Likert scale ranging from 1 (almost never) to 5 (almost always), with higher scores reflecting greater use of each strategy. Following prior studies (e.g., Garnefski & Kraaij, 2006), two composite indices were computed: one for adaptive and one for maladaptive strategies. The Hungarian version used in this study was identical to that applied in previous research (e.g., Klein et al., 2024), without modification. Internal consistency was acceptable for both higher-order dimensions (adaptive: McDonald’s *ω* = .80; maladaptive: McDonald’s *ω* = .78).

### Statistical Analysis

According to the preregistered analysis plan (see OSF: 10.17605/OSF.IO/F8WV6), we first conducted three separate GLMs to examine which psychological variables significantly predicted openness to sugar relationships. Specifically, we tested whether EMS, CERQ, and LPFS were associated with ASR scores.

In Model 1, the dependent variable was the ASR score, and the independent variables were the adaptive CERQ score, maladaptive CERQ score, and the LPFS total score.

In Model 2, the maladaptive CERQ score was used as the dependent variable, with the EMS total score entered as a predictor.

In Model 3, the LPFS total score served as the outcome variable, predicted by EMS.

Age was included as a covariate in all three models. We did not estimate a model with adaptive CERQ as the outcome, as it was not a significant predictor of ASR in Model 1.

Subsequently, we performed structural equation modeling (SEM) to assess the fit of a theoretically grounded model based on the GLM results. In this model:

- ASR was predicted by both LPFS and maladaptive CERQ scores,
- LPFS was predicted by EMS and age,
- And Maladaptive CERQ was also predicted by EMS.

All continuous variables were standardized prior to model estimation. Robust weighted least squares (WLSMV) estimation was used, appropriate for ordinal and non-normally distributed data (Bandalos, 2014).

Model fit was evaluated using multiple indices: relative chi-square (*χ*^2^/*df*), comparative fit index (CFI), Tucker–Lewis index (TLI), root-mean-square error of approximation (RMSEA), and standardized root-mean-square residual (SRMR). Acceptable model fit was defined as: *χ*^2^/*df* ≤ 3; CFI and TLI ≥ .95; RMSEA and SRMR ≤ .08 (Browne & Cudeck, 1992; Hu & Bentler, 1998).

The data supporting the findings of this study are openly available on the OSF repository (10.17605/OSF.IO/F8WV6). All statistical analyses were conducted using Jamovi version 2.5 for Windows (The jamovi project, 2024). A correlation matrix of all main study variables is presented in Supplementary Table 1.

## Results

We first tested our hypotheses concerning the predictors of openness to sugar relationships, maladaptive emotion regulation, and personality functioning. Table 1 presents the detailed statistical results of the GLM. All three models were statistically significant:

**Table 1:** Results of GLMs 1–3: The associations of openness to sugar relationships (ASR) with adaptive and maladaptive cognitive emotion regulation strategies (CERQ), level of personality functioning (LoPFS); and the association between early maladaptive schemas (EMS) and age and maladaptive CERQ and LoPFS

			95% CI					
	Variable	*B*	Lower	Upper	*β*	*df*	*t*	*p*
Model 1: DV: ASR	(Intercept)	13.90	13.17	14.63	< .001	489	37.61	< .001
	Adaptive CERQ	−0.05	−1.13	1.03	−0.005	489	−0.10	0.920
	Maladaptive CERQ	1.37	0.05	2.70	0.117	489	2.04	0.042
	LoPFS	2.02	0.67	3.39	0.164	489	2.93	0.003
Model 2: DV: Maladaptive CERQ	(Intercept)	3.07	3.02	3.12	< .001	490	118.66	< .001
	EMS	0.09	0.08	0.10	0.605	490	16.83	< .001
	age	< .001	−0.01	0.01	0.001	490	0.03	0.971
Model 3: DV: LoPFS	(Intercept)	1.32	1.28	1.36	< .001	490	67.49	< .001
	EMS	0.11	0.10	0.12	0.767	490	26.73	< .001
	age	−0.01	−0.01	−0.001	−0.065	490	−2.29	0.023

Model 1: *F*(3, 489) = 11.21, *p* < .001, *R*^2^ = .06;

Model 2: *F*(2, 490) = 141.87, *p* < .001, *R*^2^ = .36;

Model 3: *F*(2, 490) = 362.73, *p* < .001, *R*^2^ = .59.

Results indicated that ASR scores were positively associated with maladaptive CERQ and LPFS total scores. No significant association was found between ASR and adaptive CERQ scores. Furthermore, Maladaptive CERQ scores were positively associated with EMS scores, but not with age. Finally, LPFS total scores were positively associated with EMS scores and negatively associated with age.

Next, we tested the hypothesis that LPFS and maladaptive CERQ mediate the relationship between early maladaptive schemas and openness to sugar relationships (see Fig. 2 for the tested SEM model). Only significant predictors identified in the GLMs were included in the model. The structural equation model demonstrated good fit: *χ*^*2*^*(*3) = 5.57, *p* = .135, CFI = 0.994, TLI = 0.983, RMSEA = 0.042, 90% CI [0.000, 0.095], SRMR = 0.031.

For exact standardized path coefficients (*β*-values), see Fig. 2. For a complete summary of point estimates, standard errors, *β*-values, *z*-values, and *p*-values, refer to Supplementary Table 2. The model confirmed our expectations: ASR (*R*^2^ = .09) was positively predicted by both maladaptive CERQ and LPFS total scores, while maladaptive CERQ (*R*^2^ = .45) and LPFS total (*R*^2^ = .70) were positively predicted by EMS scores. Age was negatively associated with LPFS, but not significantly associated with ASR.

Taken together, the results support H1 and H2, demonstrating that both early maladaptive schemas and impairments in personality functioning are positively associated with openness to sugar relationships. In contrast, H3 was only partially supported, as maladaptive—but not adaptive—emotion regulation strategies emerged as significant predictors.

## Discussion

### Summary of the Structural Model and Main Findings

The present study examined a theoretically grounded structural model linking EMS to ASR through two key mediating mechanisms: impairments in personality functioning (LoPFS) and the use of maladaptive CERQ. The model was derived from schema theory (Young et al., 2003), the AMPD (American Psychiatric Association, 2013), and cognitive emotion regulation frameworks (Garnefski & Kraaij, 2006), and was informed by prior findings on transactional sexual attitudes and relational vulnerability (Birkás et al., 2020; Láng et al., 2021; Shareh, 2016).

The results confirmed all four preregistered hypotheses. First, openness to sugar relationships was positively associated with early maladaptive schemas (H1), suggesting that individuals with stronger cognitive-affective vulnerabilities were more accepting of transactional relational formats (Pilkington et al., 2021; Tyler, 2009). Second, impairments in personality functioning significantly predicted ASR (H2), indicating that reduced capacities for self-direction, identity integration, or intimacy may predispose individuals toward non-conventional romantic involvement (Bach & Hutsebaut, 2018; Edlund et al., 2023). Third, openness to sugar relationships was positively associated with the use of maladaptive emotion regulation strategies, but not with adaptive strategies (H3), highlighting the role of cognitive-emotional dysregulation in shaping relational preferences (Dubé et al., 2020; Zahm et al., 2021). Finally, structural equation modeling supported the proposed mediational model (H4): both personality dysfunction and maladaptive emotion regulation significantly mediated the association between EMS and ASR, confirming that early psychological vulnerabilities exert indirect effects on relational attitudes through these proximal mechanisms.

Together, these findings offer empirical support for a multidimensional psychological model of openness to sugar relationships, in which early adverse experiences and their cognitive-emotional consequences are expressed through structurally impaired personality functioning and maladaptive coping. The confirmed pathways highlight the value of integrating developmental, personality, and emotion regulation perspectives in understanding individual differences in transactional sexual attitudes (Bach & Anderson, 2020; Weiss et al., 2011).

### From Schemas to Personality Functioning: Internalized Relational Vulnerabilities

The first mediating pathway identified in our model highlights the role of impaired personality functioning as a conduit through which early maladaptive schemas influence relational attitudes. Specifically, individuals with higher EMS scores—reflecting entrenched beliefs about abandonment, emotional deprivation, or defectiveness—also showed significantly greater impairments in core domains of self- and interpersonal functioning. This association aligns with schema theory, which posits that unmet emotional needs in early life become internalized as rigid cognitive-affective structures, shaping not only momentary responses but also enduring patterns of self-organization and relational behavior (Pilkington et al., 2021; Young et al., 2003).

Impairments captured by the LPFS represent foundational disruptions in identity coherence, self-direction, empathy, and intimacy (Bach & Hutsebaut, 2018). These impairments often manifest as emotional lability, unstable goals, and difficulties forming mutual and secure connections—features that may render emotionally complex, reciprocal partnerships more difficult to navigate. As such, individuals with elevated EMS scores may come to rely on simplified, instrumental forms of intimacy, such as sugar relationships, that allow for relational contact without deep emotional investment (Láng & Birkás, 2023).

Previous research on sex workers and individuals involved in transactional intimacy has similarly documented high rates of personality dysfunction, particularly in the domains of identity diffusion and intimacy avoidance (Brüne et al., 2017; Edlund et al., 2023). Our findings extend this literature by showing that even in a non-clinical, population-based sample of women, structural impairments in personality functioning partially explain why some individuals find transactional intimacy psychologically acceptable or even preferable.

Taken together, these results support the view that early maladaptive schemas—especially those rooted in unmet attachment needs—can crystallize into broader impairments in self and relational functioning. These impairments, in turn, increase receptivity to relationship formats that minimize affective exposure and emphasize role clarity, strategic exchange, or emotional distance (Bach & Anderson, 2020; Tyler, 2009).

### Emotion Dysregulation as a Parallel Mediating Pathway

The second mediating route identified in our structural model implicates maladaptive cognitive emotion regulation as a distinct psychological mechanism through which EMS shape ASR. Specifically, higher EMS scores were strongly associated with greater use of dysfunctional regulatory strategies—such as rumination, self-blame, catastrophizing, or blaming others—which, in turn, predicted increased openness to transactional intimacy (Garnefski et al., 2001; Young et al., 2003).

This pattern is consistent with theoretical models proposing that individuals who lack effective emotional coping strategies are more likely to adopt externalizing or instrumental behaviors to manage distressing emotional states (Campbell-Sills & Barlow, 2007; Gross, 1998). In particular, cognitive distortions rooted in maladaptive schemas may amplify affective instability, increasing the salience of short-term relief or perceived control as behavioral motivators (Pilkington et al., 2021).

For women with heightened schema-based vulnerabilities, the idea of sugar relationships may be perceived as offering a more predictable or structured relational format in which emotional ambiguity and vulnerability can be minimized. Such perceptions may shape attitudinal openness, even if actual engagement in sugaring involves far more emotional complexity and unpredictability (Palomeque Recio, 2022; Scull, 2020). Rather than confronting or regulating aversive internal states through adaptive means (e.g., reappraisal, problem-solving), these individuals may engage in relational formats that afford symbolic compensation, strategic control, or psychological detachment (Bach & Anderson, 2020).

Empirical findings have linked maladaptive emotion regulation strategies to risky or avoidant sexual behaviors, especially in trauma-exposed or emotionally dysregulated populations (Dubé et al., 2020; Weiss et al., 2011; Zahm et al., 2021). Our results demonstrate that such associations extend to attitudinal receptivity toward transactional relationships in the general population, and that maladaptive regulation strategies may serve not only as correlates of emotional distress, but as affective shortcuts that shape long-term relational orientations.

Importantly, this pathway operated independently of personality functioning, indicating that emotional dysregulation constitutes a distinct mechanism—rather than a mere consequence—of structural personality impairment (Bach & Hutsebaut, 2018). Together with the LPFS-mediated route, this parallel path underscores the multifactorial nature of psychological influences on ASR and the importance of addressing both cognitive-affective schemas and regulatory habits when interpreting transactional sexual attitudes.

### Converging Pathways to Openness to Sugar Relationships

Our structural model integrates the dual mediating effects of personality dysfunction and maladaptive emotion regulation, demonstrating that these two pathways converge to explain how early cognitive-affective vulnerabilities contribute to ASR. While each mediator represents a distinct psychological domain—structural personality organization and emotion regulation habits—they jointly account for the indirect influence of EMS on relational attitudes (Bach & Hutsebaut, 2018; Garnefski & Kraaij, 2006).

This convergence supports a multidimensional conceptualization of ASR, in which transactional intimacy is not merely the product of surface-level attitudinal permissiveness or sociocultural influences (e.g., sociosexuality or short-term mating orientation; Meskó et al., 2024; 2025) but rather reflects a deeper psychological architecture. Specifically, individuals who have internalized insecure relational expectations through early maladaptive schemas may be more likely to exhibit both impaired self/other functioning and ineffective emotional coping (Pilkington et al., 2021; Tyler, 2009). These internal characteristics, in turn, predispose them toward relational formats that are less emotionally demanding, more predictable, and more instrumental in nature.

The combined mediation pattern observed in our model aligns with theoretical proposals in schema therapy and personality science, which emphasize that core schemas express themselves through multiple channels, including affective regulation strategies and broader relational functioning (Bach & Anderson, 2020; Young et al., 2003). Moreover, the fact that both mediators retained significance when modeled simultaneously highlights their complementary—not redundant—contributions to shaping ASR (Weekers et al., 2019).

These findings further suggest that sugar relationships may serve compensatory psychological functions: They offer structure, security, and symbolic value in lieu of emotional reciprocity (Edlund et al., 2023). For some individuals, such arrangements may help preserve self-coherence and protect against rejection or intimacy-based dysregulation. From this perspective, openness to sugar relationships can be interpreted not as deviant or exploitative per se, but as a functional adaptation within the constraints of an individual’s personality structure and emotional coping capacity (Bach & Anderson, 2020; Shareh, 2016).

### Broader Theoretical and Practical Implications

These findings expand upon prior research showing that short-term mating orientation is a stronger predictor of openness to sugar relationships than life history strategy (Meskó et al., 2025). While that study emphasized the evolutionary and sociosexual dimensions of ASR, the present results point to additional psychological mechanisms underlying these attitudes. Specifically, openness to sugar relationships appears to be associated not only with permissive mating strategies, but also with deeper intrapsychic vulnerabilities—such as early maladaptive schemas, impaired personality functioning, and maladaptive emotion regulation. This suggests that transactional intimacy may reflect both strategic orientation and psychological compensation. By integrating these perspectives, the current model highlights how early relational experiences and internal vulnerabilities may increase receptivity to resource-based relational formats, even in the absence of explicit life history trade-offs. It is also important to acknowledge that the notion of consent in sugar dating has been described as complex and sometimes ambiguous, with scholars noting that negotiated exchanges may obscure power asymmetries or emotional pressures (e.g., Palomeque Recio, 2022; Scull, 2020). Although examining consent dynamics falls beyond the scope of this attitudinal study, this contextual complexity should be kept in mind when interpreting receptivity to sugar relationships.

The integrative model tested in this study carries important implications for both psychological theory and applied contexts. Theoretically, our findings reinforce the value of approaching ASR not as an isolated attitudinal construct, but as a multidetermined outcome rooted in deeper personality and emotional processes. By demonstrating that early maladaptive schemas exert their influence on ASR via two distinct yet converging pathways—personality dysfunction and maladaptive emotion regulation—we extend previous models of transactional intimacy (e.g., Láng & Birkás, 2021; Meskó et al., 2025) and situate them within a broader framework of developmental psychopathology and relational functioning (Bach & Anderson, 2020; Young et al., 2003).

This perspective invites a rethinking of transactional sexual attitudes as markers of latent vulnerability rather than mere sociosexual orientation or opportunistic strategy (Shareh, 2016; Tyler, 2009). ASR may reflect a coherent, if constrained, adaptation to psychological structures characterized by identity diffusion, emotional instability, and an avoidant stance toward intimacy (Bach & Hutsebaut, 2018; Weekers et al., 2019). Accordingly, dimensional models of personality (e.g., AMPD; American Psychiatric Association, 2013) and schema-based approaches (Young et al., 2003) provide a fertile foundation for exploring how such attitudes develop and persist across the lifespan.

In clinical settings, these results suggest that individuals who endorse transactional relational preferences may benefit from therapeutic interventions targeting not only behavioral patterns but also underlying personality organization and emotion regulation. Schema therapy (Young et al., 2003), mentalization-based treatment (Bateman, 2022), and emotion-focused approaches (Elliott & Greenberg, 2021) may all be useful in addressing the internal mechanisms that make relational vulnerability more likely to be managed through instrumental exchange rather than mutual intimacy.

From a public health and psychoeducational standpoint, these findings highlight the importance of emotional competence and relational literacy in promoting healthy relationship decision-making (Collins & van Dulmen, 2020; Halpern-Meekin et al., 2013). Programs aimed at improving emotion regulation skills, fostering secure identity formation, and increasing awareness of relational dynamics could contribute to more adaptive sexual and romantic choices—particularly among young adults navigating a complex and often commodified dating landscape (Davila et al., 2017).

Finally, these insights carry implications for the broader societal discourse on sugar relationships. Rather than viewing such arrangements through moralistic or economic lenses alone, recognizing their psychological underpinnings may foster greater empathy and more nuanced understanding (Gunnarsson, 2023). A psychologically informed perspective allows for differentiation between freely chosen, strategic behavior and relational coping rooted in psychological constraint—an important distinction for both researchers and practitioners.

### Limitations and Future Directions

While the present study offers novel insights into the psychological underpinnings of openness to sugar relationships, several limitations warrant consideration. First, the cross-sectional design precludes causal inference. Although our structural model is theoretically grounded and statistically supported, it does not allow for conclusions about the temporal ordering of early maladaptive schemas, personality dysfunction, emotion regulation, and relational attitudes (Kraemer et al., 2001). Longitudinal research is needed to examine how these pathways develop over time, and to determine whether openness to transactional intimacy reflects a stable, trait-like disposition or a context-dependent coping strategy (McAdams & Olson, 2010; Widiger et al., 2019).

Second, our reliance on self-report instruments introduces potential biases, including social desirability effects, limited introspective access, and shared method variance (Conway & Lance, 2022). Although we employed validated measures and ensured anonymity, future research should adopt multi-method approaches—such as clinician-rated assessments (e.g., STiP‑5.1; Zimmermann et al., 2014), behavioral tasks, or informant reports—to enhance construct validity and capture less consciously accessible dimensions of personality and emotion regulation (Makol & De Los Reyes, 2020).

Third, although our model focused on three theoretically relevant psychological domains (EMS, LPFS, and CERQ), it omitted several important constructs—such as attachment styles, sociosexual orientation, moral evaluations, sexual self-esteem, and perceived financial stress. Future research should broaden the nomological network surrounding ASR by examining how multiple motivational, contextual, and structural variables jointly shape openness to sugar relationships (Birkás et al., 2020; Ipolyi et al., 2021; Láng et al., 2021; Meskó et al., 2024, 2025).

Another limitation concerns the use of composite scores for EMS and LPFS. While this approach improved statistical parsimony, it may have obscured meaningful heterogeneity across specific schemas or facets of personality functioning. Future studies employing latent class or profile analysis could identify subtypes of openness to sugar relationships based on distinct constellations of psychological vulnerability (Magidson & Vermunt, 2020). Likewise, psychological network analysis could illuminate the differential centrality of individual variables within personalized patterns of relational cognition (McNally, 2021; Robinaugh et al., 2020).

Finally, although we drew on research related to sex work and transactional sex to inform our theoretical model, sugar relationships differ in important ways from formal sex work in terms of structure, motivation, and cultural framing (Bernstein, 2007; Gunnarsson, 2023). Future comparative studies should investigate whether the psychological pathways identified here are unique to ASR or shared across diverse forms of transactional intimacy.

In sum, advancing this research agenda will require longitudinal, cross-cultural, and multi-method investigations to clarify how deep-seated psychological configurations influence sexual and relational preferences in contemporary social contexts. Our findings represent an important step toward such understanding by demonstrating how early vulnerabilities, personality functioning, and emotional coping converge to shape openness to transactional relationships.

### Conclusions

This study provides empirical support for a multidimensional psychological model of openness to sugar relationships, demonstrating that early maladaptive schemas shape relational attitudes through distinct yet converging pathways of impaired personality functioning and maladaptive emotion regulation. These findings suggest that ASR may serve as a compensatory adaptation grounded in psychological vulnerability, rather than reflecting mere attitudinal permissiveness or opportunistic strategy. Viewing transactional intimacy through this lens underscores the importance of developmental, personality, and regulatory processes in shaping relational preferences—and calls for more nuanced, psychologically informed approaches in both research and clinical practice.

## Supplementary Information

Below is the link to the electronic supplementary material.

Supplementary file1 (DOCX 21 KB)

## Data Availability

This study was preregistered on the Open Science Framework. All materials, de-identified data, and analysis code are available at: 10.17605/OSF.IO/F8WV6.
