# Supplementary material for: Openness to “Sugar Relationships” Reflects Personality and Emotional Vulnerabilities in a Representative Sample of Hungarian Women
Source: Arch Sex Behav. 2026 Mar 10;55(2):575–88. doi: 10.1007/s10508-025-03389-8 (PMC13048946; doi:10.1007/s10508-025-03389-8)
Supplement: Supplementary file 1 — Supplementary file1 (DOCX 21 KB) [file 10508_2025_3389_MOESM1_ESM.docx]

Openness to Sugar Relationships Reflects Personality and Emotional Vulnerabilities in a Representative Female Sample

**Supplementary Materials**

In these Supplementary Materials, we present the correlation matrix for all the variables, and the exact statistical values for the Structural Equation Model included in our study.

**Supplementary Table 1.** Pearson correlation matrix for all variables included in our study.

|  | | | | | | | | | | | | | | | |
| --- | --- | --- | --- | --- | --- | --- | --- | --- | --- | --- | --- | --- | --- | --- | --- |
|  | |  | | **ASR** | | **LPFS total** | | **Adaptive CERQ** | | **Maladaptive CERQ** | | **EMS total** | |  | |
| LPFS total |  | Pearson's r |  | 0.236 |  | — |  |  |  |  |  |  |  |  |  |
|  |  | p-value |  | < .001 |  | — |  |  |  |  |  |  |  |  |  |
| Adaptive CERQ |  | Pearson's r |  | 0.025 |  | 0.013 |  | — |  |  |  |  |  |  |  |
|  |  | p-value |  | 0.587 |  | 0.775 |  | — |  |  |  |  |  |  |  |
| Maladaptive CERQ |  | Pearson's r |  | 0.217 |  | 0.612 |  | 0.229 |  | — |  |  |  |  |  |
|  |  | p-value |  | < .001 |  | < .001 |  | < .001 |  | — |  |  |  |  |  |
| EMS total |  | Pearson's r |  | 0.338 |  | 0.770 |  | 0.070 |  | 0.606 |  | — |  |  |  |
|  |  | p-value |  | < .001 |  | < .001 |  | 0.122 |  | < .001 |  | — |  |  |  |
| Age |  | Pearson's r |  | -0.074 |  | -0.096 |  | -0.003 |  | -0.022 |  | -0.039 |  |  |  |
|  |  | p-value |  | 0.100 |  | 0.034 |  | 0.943 |  | 0.622 |  | 0.388 |  |  |  |
|  | | | | | | | | | | | | | | | |

**Supplementary Table 2.** The exact statistical values including the point estimates (B), standard errors (SE), standard estimate (ß) values, z values, and p values for the structural equation model we conducted.

|  |  |  |  | **95% CI** | |  |  |  |
| --- | --- | --- | --- | --- | --- | --- | --- | --- |
| **Dependent** | **Predictor** | **B** | **SE** | **Lower** | **Upper** | **β** | **z** | **p** |
| LoPFS total | EMS total | 0.8443 | 0.0313 | 0.78303 | 0.90553 | 0.8329 | 27.02 | < .001 |
| LoPFS total | Age | -0.0619 | 0.0279 | -0.11655 | -0.00724 | -0.0623 | -2.22 | 0.026 |
| Maladaptive CERQ | EMS total | 0.6843 | 0.0428 | 0.60030 | 0.76826 | 0.6728 | 15.97 | < .001 |
| ASR | Maladaptive CERQ | 0.1228 | 0.0611 | 0.00304 | 0.24259 | 0.1225 | 2.01 | 0.044 |
| ASR | LoPFS total | 0.2115 | 0.0583 | 0.09721 | 0.32573 | 0.2102 | 3.63 | < .001 |
| ASR | Age | -0.0493 | 0.0449 | -0.13737 | 0.03880 | -0.0493 | -1.10 | 0.273 |

Note: Openness to Sugar Relationships (ASR), Maladaptive Cognitive Emotion Regulation Strategies (CERQ), Level of Personality Functioning (LoPFS)
